# Squids use multiple escape jet patterns throughout ontogeny

**DOI:** 10.1242/bio.054585

**Published:** 2020-11-05

**Authors:** Carly A. York, Ian K. Bartol, Paul S. Krueger, Joseph T. Thompson

**Affiliations:** 1 Department of Biology, Lenoir-Rhyne University, Hickory, NC 28601, USA; 2 Department of Biological Sciences, Old Dominion University, Norfolk, VA 23529, USA; 3 Department of Mechanical Engineering, Southern Methodist University, Dallas, TX 75275, USA; 4 Department of Biology, Franklin and Marshall College, Lancaster, PA 17604, USA

**Keywords:** Squid, Escape jet, Velocimetry, Paralarvae, Propulsive efficiency

## Abstract

Throughout their lives, squids are both predators and prey for a multitude of animals, many of which are at the top of ocean food webs, making them an integral component of the trophic structure of marine ecosystems. The escape jet, which is produced by the rapid expulsion of water from the mantle cavity through a funnel, is central to a cephalopod's ability to avoid predation throughout its life. Although squid undergo morphological and behavioral changes and experience remarkably different Reynolds number regimes throughout their development, little is known about the dynamics and propulsive efficiency of escape jets throughout ontogeny. We examine the hydrodynamics and kinematics of escape jets in squid throughout ontogeny using 2D/3D velocimetry and high-speed videography. All life stages of squid produced two escape jet patterns: (1) ‘escape jet I’ characterized by short rapid pulses resulting in vortex ring formation and (2) ‘escape jet II’ characterized by long high-volume jets, often with a leading-edge vortex ring. Paralarvae exhibited higher propulsive efficiency than adult squid during escape jet ejection, and propulsive efficiency was higher for escape jet I than escape jet II in juveniles and adults*.* These results indicate that although squid undergo major ecological transitions and morphology changes from paralarvae to adults, all life stages demonstrate flexibility in escape jet responses and produce escape jets of surprisingly high propulsive efficiency.

This article has an associated First Person interview with the first author of the paper.

## Abstract (precis)

**Summary:** In this study, we examine the hydrodynamics and kinematics of escape jets in squids throughout ontogeny using 2D/3D velocimetry and high-speed videography.

## INTRODUCTION

Escape responses are used by many animals as their primary survival tactic against predation (Bullock, 1984). Typically, escape responses are characterized by extremely fast reaction times and high accelerations (Domenici et al., 2011). A number of species, including scallops (Cheng and DeMont, 1996; Cheng et al., 1996; Dadswell and Weihs, 1990), jellyfish (Daniel, 1983, 1985; Demont and Gosline, 1988; Katija et al., 2015), salps (Bone and Trueman, 2009; Madin, 1990) and the frogfish (Fish, 1987) accomplish an escape response through jet propulsion. Cephalopods, including the chambered *Nautilus*, octopuses, cuttlefishes and squids, are well known for their rapid escape jet responses. Unlike many octopuses and demersal cuttlefishes that can burrow and hide from predators, many squids reside exclusively in the water column throughout ontogeny with predators approaching them from all directions. Thus, they require highly effective escape responses for survival.

**Fig. 1.**
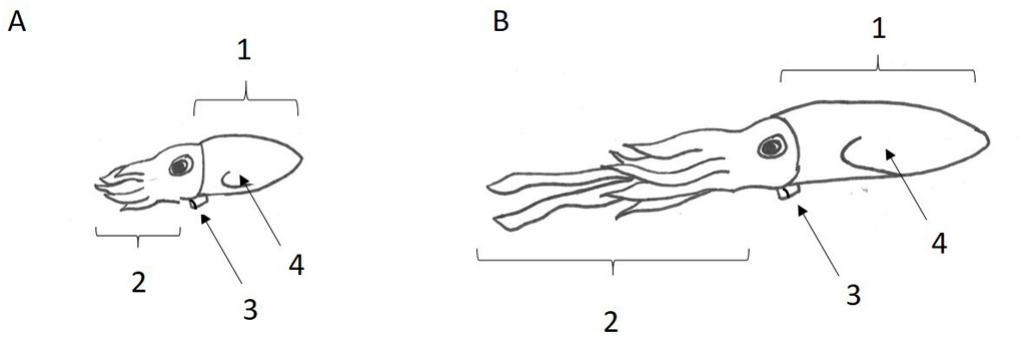
**Morphological differences between (A) paralarvae and (B) adult squids.** Major differences include paralarvae having (1) a more rounded mantle, (2) relatively smaller arms, (3) a proportionally larger funnel, and (4) rudimentary fins, when compared to adult squid.

During ontogeny, squid undergo major morphological and physiological changes that affect their locomotive abilities (Fig. 1; Boyle and Boletzky, 1996). While squid do not experience a distinct metamorphosis, and therefore do not have true larvae (they are known as paralarvae; Shea and Vecchione, 2010), hatchlings are ecologically distinct from older life history stages (Robin et al., 2014; Shea and Vecchione, 2010; Young and Harman, 1988). Moreover, relative to the adult, paralarvae have a more rounded mantle, relatively smaller arms, a proportionally larger funnel, and rudimentary fins (Boletzky, 1974; Okutani, 1987; Packard, 1969). Paralarvae also hold a proportionally greater volume of water in their cavities and have shorter thick filaments in the mantle muscles to provide jetting power (Gilly et al., 1991; Preuss et al., 1997; Thompson and Kier, 2001, 2006). Ecologically, paralarvae differ from older squid in that they cover shorter overall distances by active swimming driven mainly by the jet (Bartol et al., 2009a) and move through the water column primarily in diel vertical migrations (Boyle and Boletzky, 1996; Robin et al., 2014). Conversely, many juvenile and adult squids are capable of powerful and long distance locomotion covering significant horizontal distances, in addition to vertical migratory behavior (Boyle and Rodhouse, 2008; Gilly et al., 2006). Additionally, the physics of fluids plays an important role in the ecology of squid throughout ontogeny. Throughout ontogeny squid experience different Reynolds number (Re) flows, with Re being the ratio of inertial forces to viscous forces within a fluid (Vogel, 2003). Paralarvae reside in an intermediate Re regime (Re∼1−10^2^), where inertia and viscosity have similar relative effects on flow (Bartol et al., 2008 2009a; Thompson and Kier, 2002; Webber and O'Dor, 1986). Juvenile and adult squid operate in a higher Re regime (Re∼10^3^–10^6^), where inertial forces dominate and continuous swimming is advantageous (Bartol et al., 2009b; O'Dor, 1988).

**Fig. 2.**
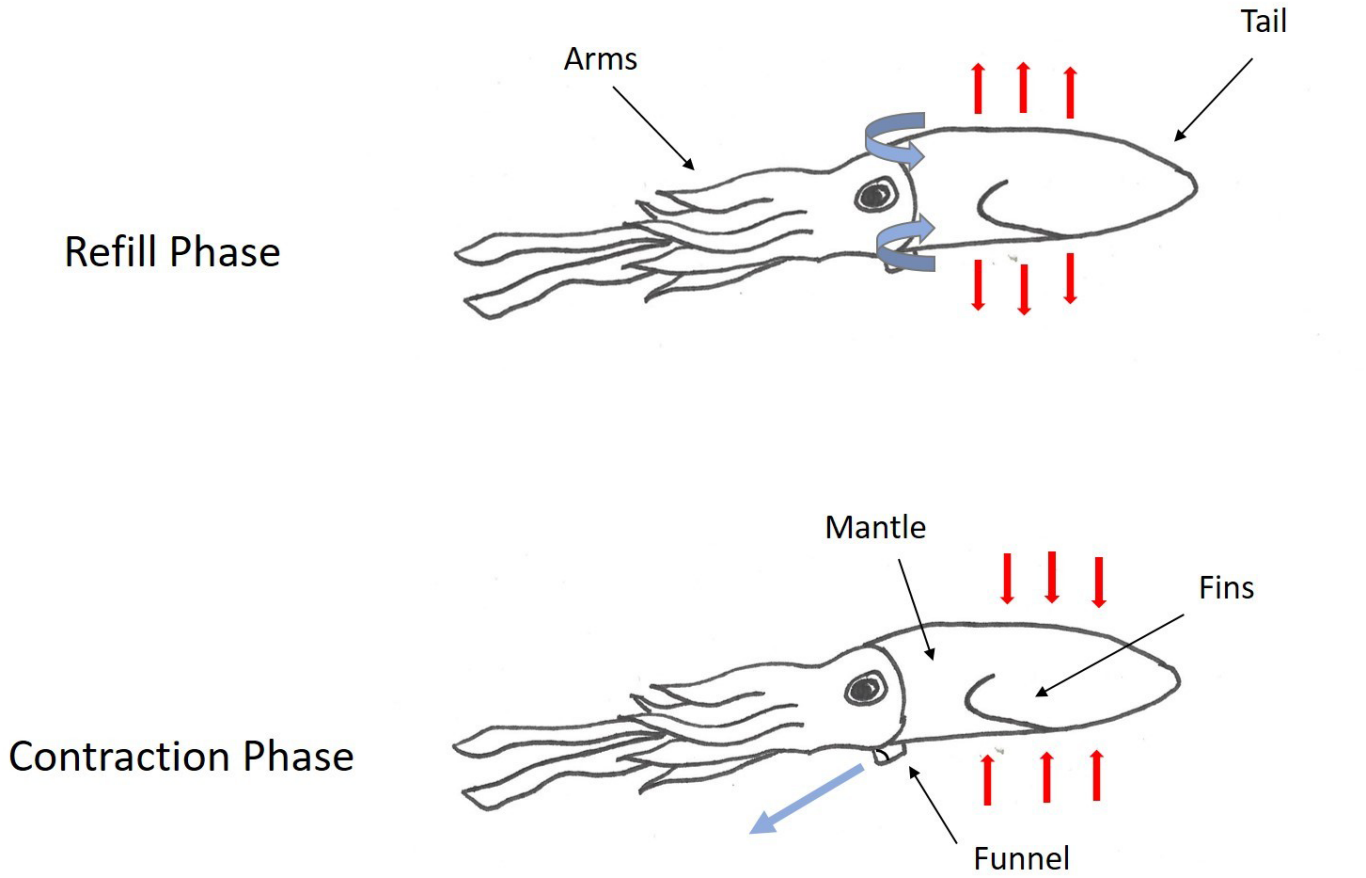
**Escape response of squids is produced by water being drawn into the mantle cavity around the sides of the head through intakes via mantle expansion produced by radial muscle contraction (refill phase), followed by muscles in the mantle then contracting to pressurize the water in the mantle cavity, resulting in the closure of the intakes.** A high velocity jet is produced when water is forcibly expelled through the funnel (contraction phase).

The jet propulsive escape response of squids is produced by the rapid expulsion of water from the mantle cavity through a funnel aperture (Fig. 2; O'Dor, 1988; Packard, 1969; Young, 1938). Water is drawn into the mantle cavity around the sides of the head through intakes via mantle expansion produced by radial muscle contraction and elastic recoil of connective tissue fibers. Circular muscles in the mantle then contract to pressurize the water in the mantle cavity, resulting in the closure of the intakes (Young, 1938). A high velocity jet is produced when water is forcibly expelled through the funnel, which has a relatively small cross-sectional area. The funnel is flexible and capable of vectoring the jet within a hemisphere below the body, which can propel the animal in various directions (Bartol et al., 2001a). Estimates of peak jet velocity range from 2.9–6.9 m/s for octopus and cuttlefish and from 6.7–11 m/s for squid (Shadwick, 1995). These high velocity jets accelerate the animal, allowing for quick evasions from oncoming predators. The nervous system of cephalopods together with their hydrostatic muscular systems (i.e. mantle and funnel) presumably allow for control of the animal's trajectory, ejected water volume, and flow speed of escape jets (Otis and Gilly, 1990), though variation in escape jetting has not been documented to date.

Although the hydrodynamics of squid escape jets have not been examined extensively, a number of studies have focused on steady routine jet propulsion in squid, with studies of both swimming energetics (Finke et al., 1996; O'Dor, 1982, 2002; Bartol et al., 2001a; O'Dor and Webber, 1991, 2011; Thompson and Kier, 2001; Webber and O'Dor, 1986; Wells and O'Dor, 1991) and hydrodynamics (O'Dor, 1988; Anderson and DeMont, 2000; Bartol et al., 2001b; Anderson and Grosenbaugh, 2005; Bartol et al., 2008, 2009a,b, 2016, 2018; Stewart et al., 2010; Staaf et al., 2014). Many of the recent hydrodynamic studies have shown that the fins and jet of squid produce complex vortical flows during steady swimming (Bartol et al., 2009a,b, 2016, 2018; Stewart et al., 2010). Additionally, studies have explored propulsive efficiency based on velocimetry measurements (Anderson and Grosenbaugh, 2005; Bartol et al., 2008, 2009a,b; Bartol et al., 2016). However, to date, no study has explicitly considered the hydrodynamics of the escape jet and how escape jetting may change throughout ontogeny.

Bartol et al. (2008, 2009b) have shown that several different types of jet flow patterns are produced by squid of different life history stages during steady rectilinear swimming. In juvenile and adult brief squid *Lolliguncula brevis*, two principal jet modes occur: (1) ‘jet mode I’, where ejected fluid rolls into an isolated vortex ring and (2) ‘jet mode II’, where ejected fluid forms into a leading vortex ring that pinches off from a long trailing jet (Bartol et al., 2008, 2009b). Jet mode I is associated with greater propulsive efficiency, lower slip (i.e. the ratio by which the jet velocity exceeds the swimming speed) and higher frequency of fin activity, while jet mode II is associated with greater time-averaged thrust (propulsive) and lift (upward) forces and is used more heavily than the first jet mode. *Doryteuthis pealeii* paralarvae produce steady jets consisting of elongated vortical ring structures but with no clear leading ring pinch-off, as is the case in jet mode II of larger size classes (Bartol et al., 2009a,b). Bartol et al. (2009a) suggested that the absence of pinch-off may be a product of either (1) viscous diffusion blurring the separation between the ring and jet or (2) vortex ring formation being preempted by viscous diffusion such that a vortical tail remains behind the ring (Bartol et al., 2009b). Bartol et al. (2008, 2009a, 2009b) found that not only do flow features differ between paralarval and juvenile/adult squid, but that paralarval squid also have higher propulsive efficiency during jet ejection than older squid when performing routine swimming.

In this study, we expand upon our knowledge of squid hydrodynamics by focusing on high velocity escape responses throughout ontogeny. Given that higher propulsive efficiencies during jet ejection occur in paralarvae compared to older squid for routine swimming, we hypothesize that similar differences will be observed for escape jetting, as the negative propulsive effects of viscosity will be reduced at higher Re while characteristics favoring improved efficiency will remain (e.g. relatively large funnel apertures, fast contraction frequencies, relatively large mantle cavity volume). Paralarvae squid that are early in their development likely have less control of jet dynamics, and consequently, we predict that older squid (juveniles and adults) will produce more hydrodynamic escape jet patterns than paralarvae. These hypotheses were tested by documenting the kinematics, velocity and vorticity of escape jets, and using direct measurements of jet wake properties (e.g. impulse, kinetic energy) to determine propulsive efficiency.

## RESULTS

**Fig. 3.**
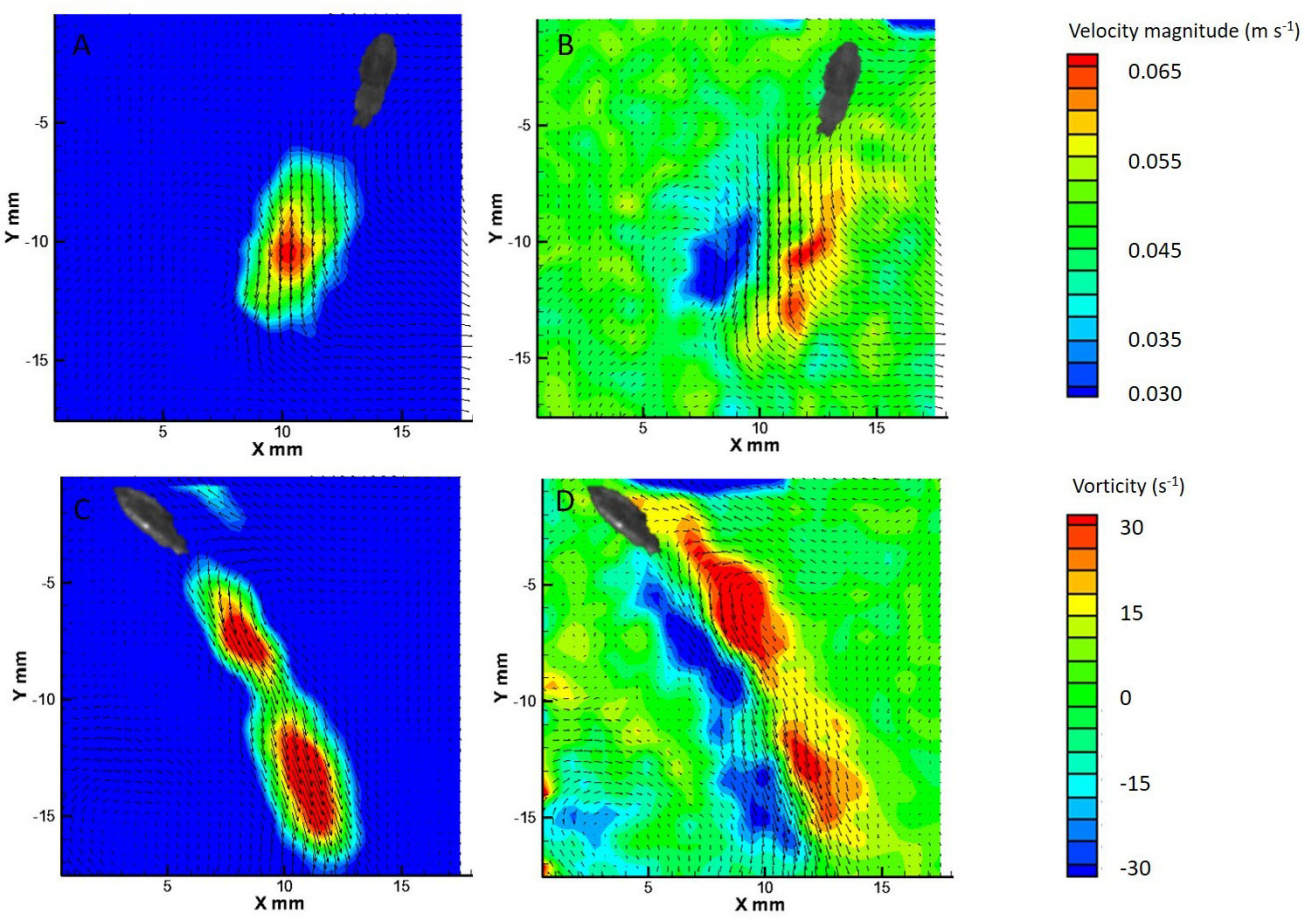
**The two hydrodynamic jet modes observed in paralarvae.** A velocity vector field of escape jet I (A) (swimming velocity=40.42 DML s^−1^) with its corresponding vorticity contour field (B) (L_ω_*/*D*_ω_*=2.77), and a velocity vector field of escape jet II (C) (swimming velocity=30.11 DML s^−1^) with its corresponding vorticity contour field (L_ω_*/*D*_ω_*=7.54).

**Fig. 4.**
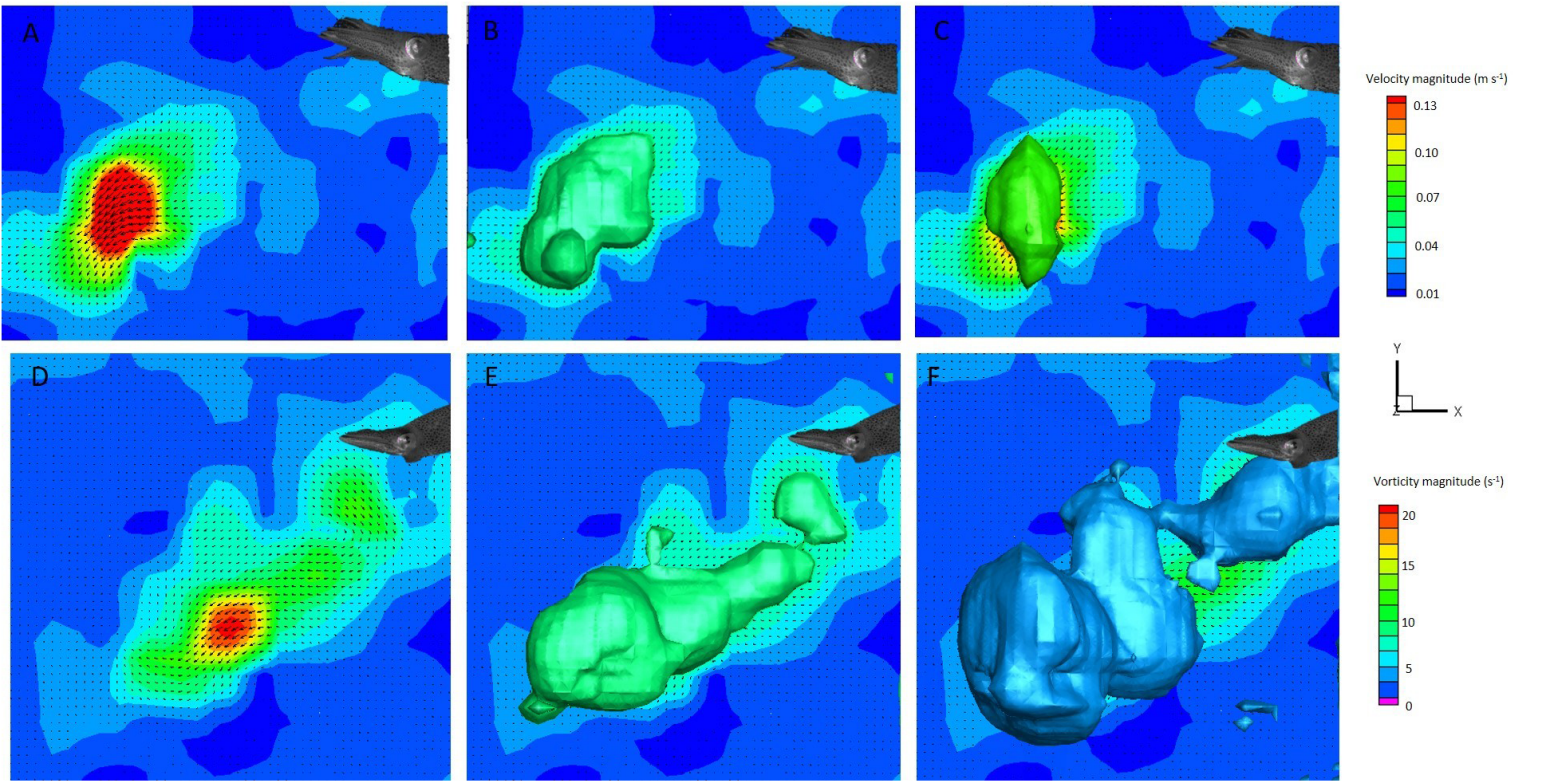
**The two hydrodynamic jet modes observed in juvenile and adults.** A 2D velocity vector field (A) (swimming velocity=2.87 DML s^−1^), velocity magnitude isosurface (B) and vorticity magnitude isosurface (C) of escape jet I (L_ω_/D_ω_=2.81) (DML=5.5 cm). A 2D velocity vector field (D) (swimming velocity=7.95 DML s^−1^), velocity magnitude isosurface (E), and vorticity magnitude isosurface (F) of escape jet II (L_ω_/D_ω_=7.53) (DML=5.30 cm).

**Fig. 5.**
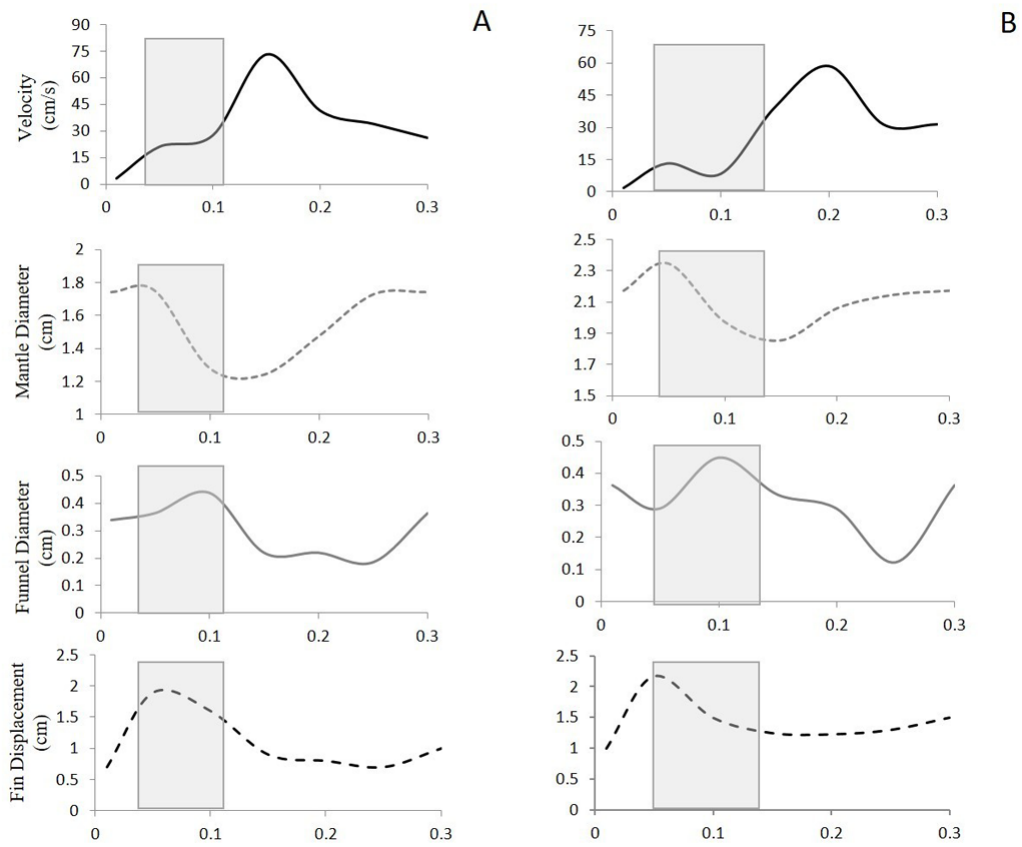
**Swimming velocity, mantle diameter, funnel diameter, and fin displacement throughout the escape response for examples of a pulsed vortex ring escape jet (escape jet I) (A) and a long escape jet (escape jet II) (B).** Adult/juvenile brief squid *L. brevis* are depicted. Mantle contraction period is highlighted.

A total of 59 escape jets were considered for this study (29 paralarval, 12 juvenile, 18 adult). Only sequences in which the animal was away from the water surface or working section walls were analyzed. All of the data presented are for tail-first escape jets. Escape jets consisted of vortical regions of variable length. Two different hydrodynamic patterns were observed: (1) escape jet I, where the jet structures consisted of spherical vortex rings with an L_ω_/D_ω_<3, and (2) escape jet II, which consisted of elongated trails of concentrated vorticity with and without discernible leading-edge vortex ring separation and an L_ω_/D_ω_>3 (Figs 3 and 4). An L_ω_/D_ω_ cut-off of three was used as jets with an L_ω_/D_ω_<3 consistently formed a spherical vortex, while an L_ω_/D_ω_>3 involved higher volume, more elongated vorticity structures (Bartol et al., 2009b). No differences were found for mean swimming velocity or acceleration between the two jet types throughout ontogeny (all *P*>0.05). Kinematic traces of escape jet I and II are characterized by a decrease in mantle diameter and funnel diameter widening at the beginning of the jet. This sequence, in addition to a single fin flap, results in peak swimming velocity (Fig. 5). Although not significant at *P*=0.05, escape jet I exhibited a trend in shorter funnel aperture periods (0.06 s) than escape jet II (0.13 s) (two-tailed *t*-test: t_5_=2.60, *P*=0.08) in juveniles and adults. Funnel aperture and mantle diameter in paralarvae could not be measured reliably due to low spatial resolution.

**Fig. 6.**
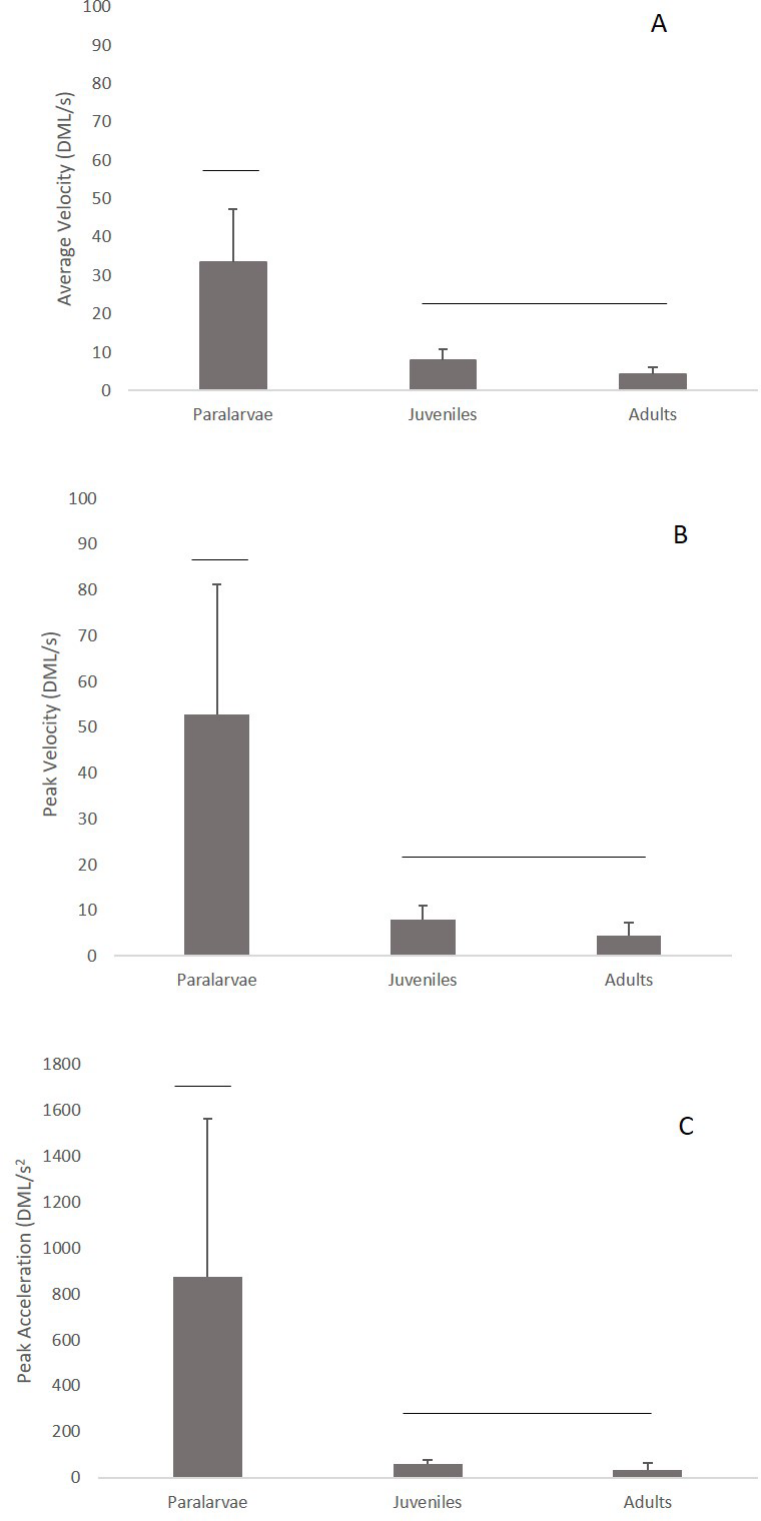
**Kinematic swimming variables throughout ontogeny.** Significant differences were found among kinematic swimming variables throughout ontogeny (MANOVA: *F*_6,86_=11.42, *P*<0.001, Wilk's _Δ_=0.31, η^2^=0.112). Average velocity (A), peak velocity (B), and peak acceleration (C) for paralarval (*N*=29), juvenile (*N*=12), and adult squid (*N*=18). DML=dorsal mantle length, s=seconds, error bars=+1 standard deviation. Lines above bars depict significant differences.

Significant differences were found among kinematic swimming variables throughout ontogeny (MANOVA: *F*_6,86_=11.42, *P*<0.001, Wilk's _Δ_=0.31, η^2^=0.112). The average velocity was significantly different among the size classes (*F*_2,59_=46.27, *P*<0.001), with the paralarvae having a higher relative average velocity (33.5±13.8 DML s^−1^; range=10.5–67.0 DML s^−1^) than both the juveniles (8.82±2.88 DML s^−1^; range=2.48–10.9 DML s^−1^; *P*<0.001) and adults (4.16±1.84 DML s^−1^; range=1.80–8.39 DMLs^−1^) (Fig. 6). The mean peak swimming velocity was also significantly different across the size classes (*F*_2,59_=27.2, *P*<0.001), where paralarvae were higher (52.8±28.3 DML s^−1^; range=12.1–120 DML s^−1^) than juveniles (9.64±3.05 DML s^−1^; range=4.81–12.0 DML s^−1^) and adults (4.56±2.84 DML s^−1^; range=1.80–11.2 DML s^−1^) (Fig. 6). Additionally, significant differences were found in peak acceleration among the three size classes (*F*_2,59_=15.36, *P*<0.001). Paralarvae had significantly higher peak acceleration (874±692 DML s^−2^; range=125–2936 DML s^−2^) than juveniles (58.3±18.1 DML s^−2^; range=33.4–78.9 DML s^−2^) and adults (35.3±26.8 DML s^−2^; range=13.4–99.0 DML s^−2^) (Fig. 6).

**Fig. 7.**
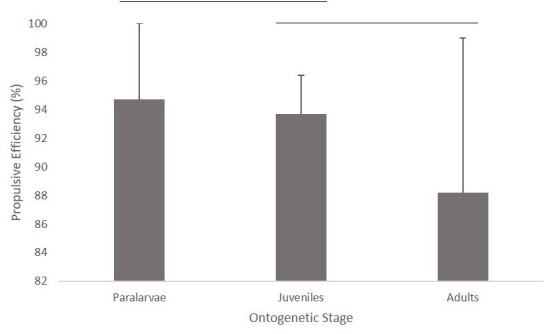
**Propulsive efficiency for paralarval (*N*=29), juvenile (*N*=12), and adult squid (*N*=18) (ANOVA: *F*_2,59_=3.94, *P*=0.025).** Error bars=+1 standard deviation. Lines above bars depict significant differences.

Significant differences in propulsive efficiency were found among the three size classes (ANOVA: *F*_2,59_=3.94, *P*=0.025). Tukey *post hoc* tests revealed that paralarvae had higher propulsive efficiency (94.7±5.3%) than the adults (88.2±11.8%) during the mantle contraction phase; however, neither paralarvae nor adults were found to have different propulsive efficiency than the juveniles (93.8±2.8) (Fig. 7). No differences in propulsive efficiency were found between the jet modes for paralarvae (two-tailed *t*-test: t_28_=0.89, *P*=0.40, Table 1). Of the hydrodynamic patterns in juveniles and adults combined, the short spherical vortex mode (escape jet I) had a higher propulsive efficiency (94.4±2.0%) than jets with a large elongated vorticity core (escape jet II) (87.3±4.3%) (two-tailed *t*-test: t_29_=2.31, *P*=0.02). Propulsive efficiency increased as mean swimming speed increased (logarithmic regression: R^2^=0.12, *P*=0.01). However, propulsive efficiency did not increase as the peak velocity increased (R^2^=0.03, *P*=0.25).

**Table 1.**
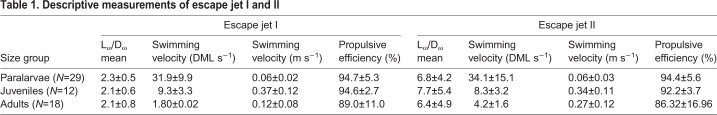
**Descriptive measurements of escape jet I and II**

## DISCUSSION

The primary survival tactic against predation is a fast and efficient escape response (Bullock, 1984). Squid from all life history stages displayed locomotive flexibility when performing an escape jet, with two distinct hydrodynamic patterns being produced (escape jet I and escape jet II). While all life stages are capable of producing a similar range of flow patterns, there are important differences in propulsive efficiency and kinematics throughout ontogeny. Although the presence of leading-edge vortex rings in longer jet pulses have been documented previously in juvenile and adult squid (Bartol et al., 2009b, 2016), they were not seen in paralarvae during routine swimming (Bartol et al., 2009a). The observance of these vortical structures in some of the escape jet II paralarval sequences in the current study suggests that leading edge pinch-off may be more apparent for stronger jets at higher Re regimes, where viscous diffusion is less prevalent. These jet wake patterns may be a product of differential recruitment of the squid's two axon systems, which control the jetting response: (1) a stereotyped giant axon system driven by a single giant axon spike, and (2) a more graded non-giant system whereby an escape jet is produced by the recruitment of both non-giant and giant axons (Otis and Gilly, 1990; Gilly et al., 1991). These two systems, in addition to mantle contraction/funnel aperture dynamics, could account for the variation observed in escape jet I and escape jet II. Irrespective of the underlying mechanism(s), our results show that different types of escape jets are possible, as opposed to one stereotyped pattern, throughout ontogeny.

Although paralarvae, juveniles, and adults exhibited similar escape jet flow patterns, differences in propulsive efficiency and kinematics throughout ontogeny were observed, likely originating from morphological and ecological differences, as well as physical constraints associated with their Re environment. Older squid Re ranged from 8000–17,000 (Re_jet_ 700–6000) while paralarvae ranged from 70–150 (Re_jet_ 20–50) in this study. The measurements of propulsive efficiency derived from properties of the jet wake (e.g. impulse, kinetic energy) indicate that paralarvae exhibit higher propulsive efficiency during jet ejection than adult squid for escape jets, which is surprising given swimming at intermediate Re is generally considered more difficult than at higher Re. The efficiency advantage of paralarvae is likely a product of several factors. Paralarvae produced a jet that had a significantly higher funnel angle relative to the horizon (76.34±13.64°) than juveniles (14.86±2.72°) and adults (20.84±8.09°) (ANOVA: *F*_2,59_=146.79, *P*<0.001), which was more aligned with their direction of motion. These results are consistent with Bartol et al. (2008, 2009a,b), who found similar angle differences. Paralarvae also have relatively larger funnel apertures (Boletzky, 1974; Packard, 1969; Thompson and Kier, 2002), faster contraction frequencies (8.6 mantle circumference lengths per second in paralarvae, versus 3.6 mantle circumference lengths per second in adults) (Thompson and Kier, 2001) and hold proportionally greater volumes of water in their mantle cavities (Gilly et al., 1991; Preuss et al., 1997; Thompson and Kier, 2001), which allow for the expulsion of large volumes of water at relatively low speeds but at high frequencies, all of which can improve propulsive efficiency (Bartol et al., 2009a).

Historically, jet propulsion at high velocities has been considered to have low efficiency compared to caudal fin propulsion typically found in fish (Alexander, 1968). However, our findings indicate that jet propulsion is a high-velocity, propulsively efficient escape mechanism throughout ontogeny in squid. The use of jet propulsion throughout ontogeny is also found in jellyfish, where *Sarsia tubulosa* has been shown to modify its swimming kinematics to maintain high propulsive efficiency (approximately 60–75%), which were calculated using similar techniques to this paper (Katija et al., 2015). Estimates for efficiency in carangiform fish are reported between 74–97% (Drucker and Lauder, 2001; Muller et al., 2001; Nauen and Lauder, 2002a,b). The propulsive efficiencies reported in our study compare with previous studies conducted on squid. Bartol et al. (2009a) found that paralarval *D. pealeii* have mean propulsive efficiencies of approximately 75% for speeds of 0.7–3.1 cm s^−1^ (Bartol et al., 2009a). The paralarval escape jet efficiency recorded here was higher (94.7%), but this is likely due to the consideration of higher swimming speeds (1.88–12.1 cm s^−1^), as propulsive efficiency tends to increase with speed in squid (Bartol et al., 2009b, 2016). Indeed Bartol et al. (2009a) found that paralarvae have deconvolved propulsive efficiencies as high as 87.5% for speeds of ∼2.5 cm s^−1^. Using models and whole-cycle efficiency calculations, Staaf et al. (2014) reported efficiencies for ommastrephid paralarvae of ∼20%. However, these results are difficult to compare directly to our results because they do not derive from direct measurements of the wake and include a refill period penalty.

As was the case here for escape jetting, Bartol et al. (2008, 2009a) found that paralarvae have higher propulsive efficiency than juveniles and adults during steady swimming. In newer 3D analyses that include both jet and fin contributions to steady swimming in *Lolliguncula brevis*, overall propulsive efficiency was 62–66% (Bartol et al., 2016, 2018). Our juvenile and adult escape jet mean propulsive efficiencies of 93.8% and 88.2%, respectively, are higher than the efficiencies above. However, when similar high-speed propulsive efficiencies are considered, the values are comparable, with 91–96% being reported in Bartol et al. (2009b, 2016). While not based on direct measures of the jet impulse and kinetic energy, propulsive efficiencies up to 93% were reported in adult *D. pealeii* when swimming at speeds >1.6 DML s^−1^ (Anderson and Grosenbaugh, 2005), which is similar to our highest recorded adult efficiency of 97%. Overall, the observed high propulsive efficiencies of high velocity squid escape jets challenge previous reports that jets are inherently inefficient (Alexander, 1968; Lighthill, 1975; Vogel, 2003).

Estimating propulsive efficiency in squids throughout ontogeny is challenging given the different Re regimes and behaviors involved. To remove the influence of gravity on propulsive efficiency in paralarvae, we considered only the exhalant phase of the jet across our ontogenetic comparisons. Although juveniles and adults generally swim along a more horizontal axis where losing ground and gravity effects are not as significant, it was important to consider propulsive efficiencies for only the propulsive phase for these life stages as well, so that fair comparisons could be made. Because the refill period involves no thrust component, it is feasible that our propulsive efficiencies are slightly overestimated. However, the relative differences among the life stages are still accurate, as the same propulsive efficiency metric was used for all comparisons.

The majority of escape sequences for all life stages included one fin flap during the beginning of mantle contraction followed by a wrapping of the fins around the mantle for the remainder of the jet cycle, a pattern commonly observed in squid during high-speed jetting (Anderson and DeMont, 2000; Bartol et al., 2018). Thrust production associated with these synchronized flaps was very low relative to the jet, particularly for paralarvae where the fin flows were barely perceivable (Bartol et al., 2008). Based on DPIV measurements of the fin wake, Stewart et al. (2010) found that the fins of *L. brevis* function as stabilizers while generating lift at low speeds and then shift to propulsors as speed increases during tail-first swimming. During arms-first swimming, the fins primarily provide lift, playing a lesser role in creating thrust (Stewart et al., 2010). Based on 3D velocimetry measurements, Bartol et al. (2016) also found that the fins of *L. brevis* sometimes act as stabilizers, producing negative thrust (drag), while consistently providing lift at low/intermediate speeds (<2.0 DML s^−1^) to counteract negative buoyancy. The lack of complex fin activity and appreciable thrust production during escape jets may be attributed to the constraints of the fin musculature and its inability to produce high forces at the high shortening velocities required for an escape jet (Kier, 1989; O'Dor and Webber, 1991). Nonetheless, every component of thrust, even limited thrust from the fins, adds to total thrust and ultimately to escape.

The paralarvae in this study showed higher average escape jet swimming velocities (33.5±13.8 DML s^−1^) than juveniles (8.8±2.9 DML s^−1^) and adults (4.2±1.8 DML s^−1^) when normalized by dorsal mantle length. The same pattern was seen in peak velocity among the three size classes, where paralarvae reached five times the peak velocity of juveniles and adults. Paralarvae also exhibited significantly greater peak acceleration than juveniles and adults. These results are consistent with the findings of Packard (1969), who found that *Loligo vulgaris* paralarvae exhibit maximum linear accelerations of 817 DML s^−2^, while juveniles reached accelerations of 316 DML s^−2^, and adults only reached 162 DML s^−2^. The ability of paralarvae to reach such high velocity and acceleration is a great advantage given the high rate of predation at this early life history stage (Boyle and Rodhouse, 2008). The average and peak velocities of the juveniles and adults reported here are lower than those reported in other kinematic studies of squid, where flow imaging was not involved (Staaf et al., 2014; York and Bartol, 2016). These differences reflect some of the challenges of collecting DDPTV data, whereby the squid are imaged in more confined experimental tanks.

While the performance metrics documented in this study are similar to those reported in fish, there are major differences found throughout ontogeny. The average peak velocity reported here in paralarvae (53 DML s^−1^) is similar to those reported in larval zebrafish when performing a C-start escape [50–65 body lengths (BL) s^−1^] (Muller et al., 2008). Juvenile and adult squid accelerations are also comparable to those found in adult fish, with numbers ranging between approximately 2–520 BL s^−2^ depending on the species and specific swimming behavior (linear acceleration, burst and coast, fast start) (Domenici and Blake, 1997; Wen et al., 2018). However, in contrast to squid, fish escape response performance improves during early development, and as larval fish grow their length-specific maximum velocity increases (Gibb et al., 2006). Both larval fish and paralarval squid experience higher Re numbers as they progress through early stages of ontogeny, but paralarval squid have morphological features (rounded mantle, proportionately larger funnels and mantle cavities, shorter thick filaments in mantle, etc.) that allow them to reach higher normalized accelerations than juveniles and adults at this early stage of development, as reported in this study. Many larval fish do not have such specific adaptations to overcome a hydrodynamic regime dominated by viscous forces, and therefore do not reach the high accelerations of adult stages, which are operating in an inertial based regime (Hale, 1996). Ecological differences may also play a role in the observed ontogenetic discrepancies between fish and squid, as many fish species (such as salmonids) are protected in nests early in development (Hale, 1999), and have a strong photonegative response during early post-hatching development, which keeps them buried and less accessible to predators (Carey and Noakes, 1981). Paralarval squid, on the other hand, generally hatch from unprotected egg mops and are immediately susceptible to predation (Boyle and Rodhouse, 2008), requiring highly effective escape systems post-hatching. Additionally, the pulsatile jet of a paralarval squid is a more ‘asymmetric’ form of propulsion than the oscillatory mechanisms of larval fish, and it may simply be more efficient at the Re experienced by animals at these early life stages (Bartol et al., 2009a). Squid are not susceptible to the scallop theorem (i.e. that time reversible motion such as oscillatory motion produces no net locomotion as Re becomes smaller; Purcell, 1977) because their anatomy includes check valves. Flapping, however, is time-reversible motion and so fish will not be able to propel themselves if they are too small. From this perspective, small squid might be expected to be relatively better swimmers than small fish (Bartol et al., 2009a; Lauga and Bartolo, 2008).

In this study, we determined that squid have flexibility in escape responses, which was evident by the observation of two different escape jet modes throughout ontogeny. Escape jet I is more efficient in juveniles and adults and may be the mode used when a threat is not eminent. Escape jet II is less efficient than escape jet I and may be used when a predatory attack is unavoidable, making a rapid escape integral for survival. Having high propulsive efficiency and the ability to swim quickly are key advantages for squid as they escape oncoming predators. Throughout all life history stages, squids are prey targets for many marine predators, including fish, marine mammals, sea birds and even other cephalopods, making them an integral component of marine food webs (Clarke, 1996; Mather, 2010; Piatkowski et al., 2001; Wood et al., 2008). Therefore, it is vital that they have an effective response to predation. When faced with an oncoming predator, the escape response often consists of several sequential escape jets to move away from the predator. Thus, there is a benefit to having high efficiency for each escape jet within a long chain of responses, as it reduces overall energy expenditure. Considering that carangiform fish range in swimming efficiencies from 74–97% (Drucker and Lauder, 2001; Muller et al., 2001; Nauen and Lauder, 2002a,b), having high propulsive efficiencies (89–95%) may confer advantages to squid and improve their success in avoiding predator attacks. Indeed, squid not only perform sequential escape jets for each interaction but also have lots of daily interactions with predators, making a highly efficient response essential for survival. The results of this study indicate that squid show locomotive flexibility and are extremely good at producing high velocity and highly efficient escape jets even in the earliest phases of life. With the fossil record of cephalopods dating back 500 million years, the evolution of this predator evasion strategy has allowed these animals to thrive and become a crucial component of our marine ecosystems (Hanlon and Messenger, 1996).

## MATERIALS AND METHODS

### Animals and maintenance

Paralarval *D. pealeii* Lesueur [dorsal mantle length (DML)=0.18 cm] and juvenile (DML=3.0–5.0 cm) and adult *L. brevis* Blainville (DML=5.1–7.0 cm) were used for this research. Paralarval *D. pealeii* are comparable to paralarval *L. brevis*, which are extremely difficult to obtain, because both species have similar body size, fin size and shape, and ecological niches during early ontogenetic stages (Bartol et al., 2008). *Doryteuthis pealeii* eggs were obtained from the Marine Biological Laboratory, Woods Hole, MA, USA, and maintained in floating buckets with mesh openings within a recirculating seawater system at a salinity of 30–32‰ and at temperatures of 19–24°C until hatching. Once the eggs hatched, the paralarvae were separated so that their ages could be tracked. A total of 170 paralarvae were used in experimental trials. *Lolliguncula brevis* used in this project were captured by otter trawl in Wachapreague, VA, USA. Trawls were conducted in August, September and October as the catch probabilities are highest in these months (Bartol et al., 2002). After capture, squid were transferred to a 114 l, circular holding tank (Angler Livewells, Aquatic Eco-Systems, Inc., Apopka, FL, USA) fitted with a portable battery powered aerator (Model B-3, Marine Metal Products Co., Inc., Clearwater, FL, USA) for transport to the lab. Squid were maintained in 450-gallon seawater systems with several forms of filtration (e.g. BioBalls, protein skimmers, ozone filtration, etc.). Seawater was maintained at temperatures and salinities equivalent to those of the capture sites (19–22°C; 30–35‰). A moderate current flow was maintained to promote active swimming and squid were fed a diet of live *Palaemonetes pugio* and *Fundulus heteroclitos* as suggested by Hanlon et al. (Hanlon, 1990; Hanlon et al., 1983). Squid were allowed to acclimate for at least 24 h prior to experimental trials. Only animals that appeared healthy and that exhibited normal behaviors were used, for a total of 22 juveniles and 26 adults.

### DPIV experiments

Digital particle image velocimetry (DPIV) was used to collect 2D hydrodynamic data from paralarval squid. We provide a general description of the approaches below, and refer the reader to Bartol et al. (2009a) for more detailed information. For paralarval experiments, three to five squid were added to a 4.0×6.0×2.5 cm chamber filled with seawater (19–24°C; 30–32‰) seeded with neutrally buoyant silver-coated glass spheres (mean diameter=14 µm, Potters Industries, Valley Forge, PA, USA), which were illuminated within a 1 mm thick laser sheet using a ND:YAG dual pulsed laser (wavelength=532 nm, intensity=350 mJ pulse^−1^; LABest Optronics, Beijing, China). A UNIQ UP-1830CL video camera (1024×1024 pixel resolution; paired images collected at 15 Hz; Uniq Vision, Inc., Santa Clara, CA, USA) outfitted with a VZM 450i zoom lens (Edmund Optics, Barrington, NJ, USA) and an optical filter allowing only 532 nm wavelengths was synchronized with laser pulses and used for data collection (time separation between paired images, ΔT, was 1–4 ms). For analysis of the DPIV data, each image was subdivided into a matrix of 32×32 pixel interrogation windows. Using a 16 pixel offset, cross-correlation was used to determine the particle displacement within interrogation windows comprising the paired images. These cross correlations were performed using *Pixelflow*™ (FG Group LLC, San Marino, CA, USA) (Willert and Gharib, 1991) and INSIGHT 4G v. 11 (TSI, Inc., Shoreview, MN, USA) software. Particle shifts that were three pixels greater than their neighbors (Pixelflow™) or local median velocity (INSIGHT) were removed as outliers and the data were smoothed to remove high frequency fluctuations. Using the software above, velocity vector (flow speed and direction) and vorticity (local rotation of the fluid) fields were determined. Deconvolution of all paralarval velocity fields was employed to account for depth averaging within the laser sheet, as the funnel of the squid was similar in size to the laser sheet thickness. Details of the deconvolution approach may be found in Bartol et al. (2009a). The magnitude of the jet impulse (**I**) and the excess kinetic energy of the jet (*E*) were computed from:(1)
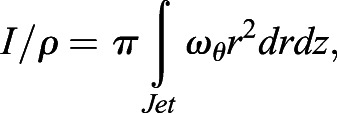
(2)
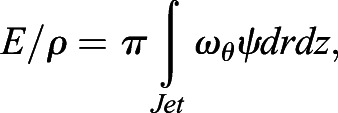
where ω_θ_ is the azimuthal component of vorticity, *r* is the radial coordinate, *z* is the longitudinal coordinate along the jet axis, *ψ* is the Stokes stream function, and *ρ* is the fluid density. The area integrals were computed using a 2D version of the trapezoidal rule. Only motion produced during jet ejection was considered because paralarvae tend to sink rapidly during refilling and work done by the propulsive system, not work done by gravity, was of interest. Therefore, propulsive efficiency (*η*_*p*_) was calculated using the equation:(3)
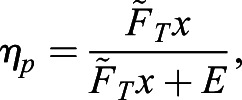
where 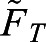 is the jet thrust time-averaged over the mantle contraction and *x* is the animal displacement during mantle contraction. 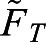
was determined by dividing the impulse component in the direction of animal displacement by the mantle-contraction period. The impulse and excess kinetic energy were computed for the frame within the sequence that captured the most complete jet. The length of the jet (*L*_*ω*_) was computed based on the extent over which the jet vorticity field was ≥20% of maximum vorticity, and jet diameter (*D*_*ω*_) was the distance (perpendicular to the jet centerline) between vorticity cores, regions where vorticity was ≥90% of peak jet vorticity. The *L*_*ω*_/*D*_*ω*_ ratio is an important metric for jet performance as it linked to the physical limit of vortex ring formation, propulsive performance, and thrust augmentation (Gharib et al., 1998; Krueger and Gharib, 2003; Bartol et al., 2009b).

### DDPTV experiments

Defocusing digital particle tracking velocimetry (DDPTV) data were collected for juvenile/adult size classes. Again, we provide a short description of the approach below and refer the reader to Bartol et al. (2016) for more detailed information. Experiments were conducted in a water tunnel [Model 502(s), Engineering Laboratory Design, Lake City, MN, USA] filled with seawater containing light-reflective particles (polyamide, 50 μm, Dantec Dynamics, Skovlunde, Denmark). The squid were allowed to acclimate for at least 5 min in the water tunnel under low flow conditions (<3 cm s^−1^), after which they were exposed to a range of flow velocities until they swam steadily against oncoming flow. The seeding particles were illuminated with the pulsed laser described above with the beam expanded to illuminate the volume of the water tunnel test section, and a V3V-8000 probe and INSIGHT 4G V3V software (TSI, Inc., Shoreview, MN, USA) were used to collect paired DDPTV images of flows around the squid at 7 Hz with ΔT=2 ms. Optical filters allowing only 532 nm wavelengths were used with the probe. In many sequences, firing the dual lasers following extended periods of steady swimming served as the trigger for escape jetting. However, in some sequences, the squid did not respond to the initial laser pulses, but did exhibit an escape response within an extended laser firing sequence. All of the juvenile/adult escape jets presented in this study occurred while the squid swam against free-stream flow, i.e. no escape jet was initiated from a resting start.

Approximately 75,000–125,000 particles were identified in each DDPTV image with triplet yields (matches of particles among the three cameras in the probe) of ∼50–60%. A relaxation method for particle tracking (Pereira et al., 2006) was used to obtain 18,000–25,000 particle vectors in the imaging volume. For interpolating the velocity vectors onto a regular grid, Gaussian weighted interpolation was used with a voxel size of 16 mm on each side, percentage overlap of 75%, and smoothing factor of 1.5. Impulse (**I**) associated with the vortical 3D flow was computed from:(4)
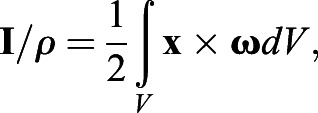
where **x** is the position vector, 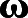 is the vorticity vector (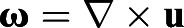, where 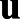 is the velocity vector), *ρ* is the fluid density, and the integral is computed over the volume of the vortex (Saffman, 1992). Excess kinetic energy (*E*) was computed from:(5)
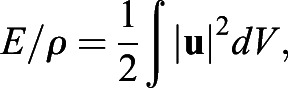
where 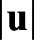 is the velocity magnitude. Bartol et al. (2016) provides further detail for these calculations. *L*_*ω*_*,* *D*_*ω*_, and 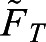 were computed similarly to the approaches described for paralarvae, and *η*_*p*_ was computed using Eqn. (3) above.

### Kinematic measurements

Video of the squid was collected simultaneously with the DPIV and DDPTV data using two high-speed DALSA Falcon video cameras (1400×1200 pixels, 100 fps, Teledyne Dalsa, Inc., Waterloo, Ontario, Canada). To prevent overexposure of frames from the laser, each high-speed camera was fitted with a filter to block 532±5 nm wavelengths. For DPIV experiments, the cameras collected images from dorsal and lateral perspectives; for DDPTV experiments the cameras collected images from ventral and lateral perspectives. A series of two to four, 500-watt halogen lights, equipped with optical filters having low transmission at 532 nm, provided illumination.

Frame-by-frame position tracking of the squid body features was accomplished using DLTdv digitizing software (Hedrick, 2008). Video was calibrated using rulers placed in the viewing chambers, allowing conversion of pixels to centimeters. Only those sequences in which the squid swam orthogonally to the longitudinal axis of the laterally positioned camera were considered, with the animal position in the z-coordinate (depth) being determined using footage from the dorsal and ventral cameras for DPIV and DDPTV applications, respectively. Moreover, animal rotation was minimal in the selected sequences, allowing for frame-by-frame tracking of landmarks on the squid. Six points were continuously tracked on the squid: (1) one eye, (2) the most anterior point of the funnel opening, (3) the most posterior point of the funnel opening, (4) dorsal edge of the thickest point of the mantle, (5) ventral edge of the thickest point of the mantle, and (6) the tip of the fin at maximum span. The tracked points were used to determine the following kinematic variables: (1) mantle diameter changes, (2) contraction and refill periods, (3) funnel angle, (4) mean velocity, (5) peak velocity, (6) peak acceleration, (7) displacement of the fins, and (8) diameter of the funnel. Due to low image resolution, kinematic variable 8 (funnel diameter) could not be determined reliably for paralarvae and thus was not considered. Swimming velocities of juveniles and adults were determined by measuring net displacement along the x-axis over complete jet cycles divided by the cycle period and adding this to the background water tunnel speed. Swimming velocities of paralarvae were determined by dividing net displacement along the path of travel over complete jet cycles by the jet cycle period. It was not necessary to correct for background flow in paralarvae trials because these experiments were performed in stationary water as opposed to a water tunnel. Using a MATLAB routine developed in-house, squid acceleration, velocity, and mantle diameter were calculated and smoothed with a fourth order Butterworth filter using a cutoff frequency of 4 Hz, which worked well for the current dataset, providing an optimal balance between excess noise and over-smoothing.

### Statistical analysis

Statistical analysis was performed in SPSS (v.18 SPSS Inc., Chicago, IL, USA). All data were tested for normality using Shapiro-Wilk tests. Multivariate analysis of variance (MANOVA) was performed to compare kinematic swimming variables in paralarvae, juveniles and adult squid. Follow-up ANOVAs for significant variables were performed, with Tukey *post hoc* tests used for multiple comparisons (SPSS). Two-tailed *t*-tests were used to compare kinematic variables between escape jets I and II. ANOVAS were used to compare propulsive efficiency among the ontogenetic stages, with subsequent Tukey *post hoc* tests for comparisons. Logarithmic regressions were performed to analyze propulsive efficiency and swimming speed. All means are presented with standard deviation, unless otherwise noted.
